# Unstable housing and hepatitis C incidence among injection drug users in a Canadian setting

**DOI:** 10.1186/1471-2458-9-270

**Published:** 2009-07-29

**Authors:** Christina Kim, Thomas Kerr, Kathy Li, Ruth Zhang, Mark W Tyndall, Julio SG Montaner, Evan Wood

**Affiliations:** 1 British Columbia Centre for Excellence in HIV/AIDS, St. Paul's Hospital, Vancouver, Canada; 2 Department of Medicine, University of British Columbia, Vancouver, Canada

## Abstract

**Background:**

There has emerged growing recognition of the link between housing and health. Since Vancouver, Canada has had increasing concerns with homelessness brought about by urban renewal in the lead-up to the 2010 Winter Olympic Games, we evaluated hepatitis C virus (HCV) incidence among injection drug users (IDU) with and without stable housing.

**Methods:**

Data were derived from a collaboration between two prospective cohort studies of IDU in Vancouver, Canada. Using Cox Proportional Hazards regression, we compared HCV incidence among participants with and without stable housing, and determined independent predictors of HCV incidence.

**Results:**

Overall, 3074 individuals were recruited between May 1996 and July 2007, among whom 2541 (82.7%) were baseline HCV-infected. Among the 533 (17.3%) individuals who were not HCV-infected at baseline, 147 tested HCV antibody-positive during follow-up, for an incidence density of 16.89 (95% confidence interval: 14.76 – 19.32) per 100 person-years. In a multivariate Cox regression model, unstable housing remained independently associated with HCV infection (relative hazard = 1.47 (1.02 – 2.13).

**Conclusion:**

HCV prevalence and incidence are high in this setting and were associated with unstable housing. Efforts to protect existing low-income housing and improve access to housing may help to reduce HCV incidence.

## Background

The World Health Organization estimates that approximately 170 million people, or 3% of the world's population, are infected with the hepatitis C virus (HCV). There are presently an estimated 3 to 4 million new infections per year [1], with illicit injection drug use being the major risk factor for HCV. In North America and Europe, the majority of HCV infections are associated with injection drug use [1]. In some Western European countries, more than 90% of injection drug users (IDU) have been found to be HCV-positive, whereas in China prevalence ranges from 34% to 93%, and similar prevalence has been reported among North American communities of IDU, with a range of 30% to 90% [2-4].

Among IDU, there are several known associations with new HCV infection, including older age, new onset of injection drug use, sharing of syringes, engaging in risky sexual behavior or prostitution, and frequent injection of cocaine [2,4]. Although interventions such as sterile syringe exchange programs have been shown to decrease new HIV infection, the results have been inconsistent when it comes to prevention of HCV infection in IDU [4-7]. There are several explanations for why these interventions have not been consistently successful in reducing new HCV infections. HCV is thought to be transmissible not only through syringes, but also through other drug paraphernalia such as "cookers" and filters [8]. Risk of hepatitis C infection is also closely linked to social and environmental factors. One study focusing on IDU perceptions of HCV risk found perceptions among IDU that HCV is ubiquitous, less serious than HIV infection, and associated with a lack of hygiene [9]. Environmental factors such as homelessness, fear of policing and arrest, and oppressive relationships were also perceived to increase HCV risk, as unstable environments disrupt individual capacity to make risk-reducing decisions [9].

Although a lack of stable housing has been linked with IDU taking part in high-risk, erratic, and unhygienic injecting practices [10], the relationship between unstable housing and HCV incidence has not been fully explored. A previous study looked at factors associated with HCV seroconversion amongst Vancouver IDU [11], however, unstable housing has not been previously described as a risk factor among long term cohorts of IDU. This is an important question in our setting (Vancouver, Canada) because there has been a loss of low-income housing and an increase in homelessness as a result of urban renewal in anticipation of the 2010 Winter Olympic Games [12]. Therefore, we sought to examine rates of HCV infection and the possible link to unstable housing among injection drug users in this Canadian setting.

## Methods

For the present study, we pooled data from participants being followed in two well characterized cohorts of IDU in Vancouver, Canada. The Vancouver Injection Drug Users Study (VIDUS) is an ongoing open prospective cohort study that was based on snowball sampling methods and outreach efforts at local services for IDU, primarily the city's needle exchange program [13]. The Scientific Evaluation of Supervised Injecting (SEOSI) cohort is an ongoing open prospective cohort study that was based on random sampling methods from the city's supervised injecting facility [14]. Both cohorts have been described in detail previously [15,16], and as previously outlined [14], the follow-up procedures and questionnaire items used for this analysis were identical in both studies to allow for the merging of data sets. In brief, the studies are observational in nature and, aside from pre- and post-HIV test counseling, no intervention is involved. Rather, both studies involve individuals providing a venous blood sample and responding to an interviewer-administered questionnaire at baseline and on a semi-annual basis, and services such as methadone and needle exchange are independent of the study. The cohorts receive annual ethical approval from the University of British Columbia/Providence Healthcare Research Ethics Board and are conducted in a fashion consistent with other prospective cohort studies of IDU. Participants were eligible for the present study if they were recruited between May 1996 and July 2007.

In this study we compared HCV prevalence levels at the time of recruitment and subsequent HCV incidence rates among participants with and without stable housing. As previously [17,18], stable housing was defined as living in an apartment or house at the time of interview, and unstable housing was defined as living in a single room occupancy hotel, shelter, recovery or transition house, jail, on the street, or having no fixed address. Housing status was a time-updated variable that was reassessed at each semi-annual follow-up visit.

All individuals recruited during the study period were eligible for the analysis of baseline HCV prevalence, whereas individuals who were baseline HCV-negative and who had at least one follow-up visit (to re-test for HCV infection) were eligible for the analysis of HCV incidence. To avoid duplication, any individual who was recruited into both cohorts was retained only in the cohort into which they were first enrolled.

The statistical analyses of HCV incidence were undertaken using an *a priori *defined statistical protocol, as follows. First, baseline characteristics of study participants stratified by housing (stable vs. unstable) were examined to evaluate potential baseline differences between these populations. Variables considered included: gender; age; ethnicity (Aboriginal vs. non-Aboriginal); residency in Vancouver's HIV epicenter known as the Downtown Eastside (DTES); use of methadone; sex trade involvement; daily heroin injection; daily cocaine injection; daily methamphetamine injection; daily crack smoking; used syringe borrowing; unsafe sex (defined as vaginal or anal sex without a condom); incarceration (defined as being in detention, jail, or prison). All variable definitions were identical to earlier studies and most heroin in Vancouver is believed to be from Asia, primarily Burma [13,19]. All behavioral variables were in reference to the prior six months, except for methadone use, which referred to current use. Pearson's chi-squared test was used to compare categorical variables, and continuous variables were compared using the Wilcoxon rank sum test.

We calculated the incidence density of HCV infection among participants with and without stable housing. As previously [20], the date of HCV seroconversion was estimated using the midpoint between the last negative and the first positive antibody test results. Participants remaining persistently HCV seronegative were censored at the time of their most recent available HCV antibody test result prior to December 2005.

We also calculated the unadjusted and adjusted relative hazards of HCV seroconversion using Cox proportional hazards regression. Here, all behavioral variables were treated as time-updated covariates based on semi-annual follow-up data. For the multivariate model, a fixed model was built that adjusted for all variables described above that were statistically associated with HCV seroconversion in unadjusted analyses. Analyses were conducted using SAS 9.1 (Cary, NC); the threshold for statistical significance was set at *p *< 0.05. All *p*-values were two-sided.

## Results

During the study period, 3074 unique individuals were recruited into either VIDUS or SEOSI, among whom 1973 (64.2%) reported living in unstable housing at baseline. Of the 3074 recruited individuals, 980 (31.9%) were female and 730 (23.8%) identified themselves as Aboriginal. Overall, the baseline HCV prevalence was 82.7%. In VIDUS, the baseline HCV prevalence was higher among those with unstable housing at baseline (84.0% vs. 78.8%, *p *= 0.004); however, in SEOSI, baseline HCV prevalence was no different among those with unstable housing at baseline (86.6% vs. 83.2%, *p *= 0.119; combined cohort: 84.3% vs. 79.7%, *p *= 0.001). At baseline, 533 participants were HCV-negative, and 390 (73.2%) of these had at least one follow-up visit and were therefore included in the analysis of HCV incidence. Among the 1101 individuals not in unstable housing at baseline, 693 (62.9%) subsequently reported being in unstable housing at least once during follow-up, whereas among the 1973 individuals in unstable housing at baseline, 1217 (61.7%) subsequently reported being in stable housing at least once.

Table 1 shows the demographic and risk behavior profile of baseline HCV-negative participants stratified by stable versus unstable housing. As shown here, participants with unstable housing were more likely to be Aboriginal (*p *= 0.030), to reside in the Downtown Eastside (*p *< 0.001), to be sex-trade involved (*p *= 0.014), and to inject methamphetamine daily (*p *= 0.014). Participants with unstable housing were less likely to report use of methadone (*p *= 0.014), to report syringe sharing (*p *= 0.027) and to use heroin daily (*p *= 0.013). There were no significant differences based on gender, incarceration within the last 6 months, daily cocaine use, daily crack smoking, and unsafe sexual practices.

**Table 1:** Baseline demographic characteristics of HCV-negative IDU stratified by unstable housing

**Characteristic**	**Stable Housing *n = 175 *(%)**	**Unstable Housing *n = 215* (%)**	**Odds Ratio (95% CI)**	***p*-value**
**Gender**				
Male	122 (69.71)	152 (70.70)		
Female	53 (30.29)	63 (29.30)	0.95 (0.62 – 1.48)	0.833
**Age**				
Median (IQR)	28 (22 – 35)	28 (23 – 40)	1.01 (0.99 – 1.03)	0.268
**Aboriginal ethnicity**				
No	153 (87.43)	170 (79.07)		
Yes	22 (12.57)	45 (20.93)	1.84 (1.06 – 3.21)	0.030
DTES residence*				
No	113 (64.57)	61 (28.37)		
Yes	62 (35.43)	154 (71.63)	4.60 (3.00 – 7.06)	<0.001
**On methadone**^†^				
No	154 (88.00)	204 (94.88)		
Yes	21 (12.00)	11 (5.12)	0.40 (0.19 – 0.84)	0.014
**Incarceration**				
No	92 (77.97)	123 (71.51)		
Yes	26 (22.03)	49 (28.49)	1.41 (0.82 – 2.44)	0.218
Sex trade involved*				
No	146 (83.43)	157 (73.02)		
Yes	29 (16.57)	58 (26.98)	1.86 (1.13 – 3.06)	0.014
Syringe sharing*				
No	105 (60.00)	152 (70.70)		
Yes	70 (40.00)	63 (29.30)	0.62 (0.41 – 0.95)	0.027
Heroin use*				
Less than daily	94 (53.71)	142 (66.05)		
Daily use	81 (46.29)	73 (33.95)	0.60 (0.40 – 0.90)	0.013
Cocaine use*				
Less than daily	146 (83.43)	167 (77.67)		
Daily use	29 (16.57)	48 (22.33)	1.45 (0.87 – 2.41)	0.156
**Crack smoking**				
Less than daily	147 (84.00)	168 (78.14)		
Daily use	28 (16.00)	47 (21.86)	1.47 (0.88 – 2.46)	0.144
**Methamphetamine injection**				
Less than daily	156 (89.14)	172 (80.00)		
Daily	19 (10.86)	43 (20.00)	2.05 (1.15 – 3.67)	0.014
Unsafe sex**				
No	100 (57.14)	133 (61.86)		
Yes	75 (42.86)	82 (38.14)	0.82 (0.55 – 1.23)	0.345

Note: IDU = injection drug user; DTES = Downtown Eastside. *Indicates behavior during the six-month period prior to the baseline interview. ^†^Indicates current use. **Unsafe sex was defined as vaginal or anal sex without a condom.

Among the 533 (17.3%) individuals who were not HCV-infected at baseline, 147 tested HCV antibody-positive during follow-up, for an incidence density of 16.89 (95% confidence interval [CI]: 14.76 – 19.32) per 100 person-years. Table 2 shows the results of the unadjusted and adjusted Cox proportional hazard regression analyses of the time to HCV infection for HCV risk behaviors and demographic characteristics. As shown here, in unadjusted Cox regression analyses, the relative hazard [RH] of HCV seroconversion for those with unstable versus stable housing was 1.71 (95% CI: 1.22 – 2.39; *p *= 0.002). After adjusting for all variables associated with the time to HCV infection in univariate analyses, as well as for cohort of initial recruitment, the RH of HCV infection was 1.47 (95% CI: 1.02 – 2.13; *p *= 0.041) for participants with unstable versus stable housing. Other variables associated with HCV seroconversion in the adjusted Cox regression analyses included syringe sharing, with a RH of 1.57 (95% CI: 1.08 = 2.29; *p *= 0.020); daily heroin injection with a RH of 1.61 (95% CI: 1.13 – 2.28; *p *= 0.008); and daily cocaine injection with a RH of 1.48 (95% CI; 1.00 – 2.19; *p *= 0.049).

**Table 2:** Univariate and multivariate^† ^Cox proportional hazard analyses of the time to HCV infection among 390 injection drug users.

	**Unadjusted Relative Hazard (RH)**			Adjusted** Relative Hazard (RH)		
		
**Variable**	**RH**	**(95% CI)**	***p*-value**	**RH**	**(95% CI)**	***p*-value**
Housing*						
(unstable vs. stable)	1.71	(1.22 – 2.39)	0.002	1.47	(1.02 – 2.13)	0.041
Syringe sharing*						
(yes vs. no)	1.84	(1.27 – 2.65)	0.001	1.57	(1.08 – 2.29)	0.020
Heroin injection*						
(daily vs. not daily)	2.00	(1.44 – 2.79)	<0.001	1.61	(1.13 – 2.28)	0.008
Cocaine injection*						
(daily vs. not daily)	1.95	(1.34 – 2.84)	< 0.001	1.48	(1.00 – 2.19)	0.049

†Only significant variables are shown above. Model was adjusted for initial cohort of recruitment (*p *> 0.05) and all variables significant in unadjusted analyses: gender, sex-trade, DTES residence (all *p *> 0.05 after adjustment). *Data were time-updated semi-annually and behaviors refer to activities in the last six months.

## Discussion

The present study demonstrated a high prevalence and incidence of HCV among IDU in a Canadian setting. Among those individuals who were not HCV-infected at baseline, the incidence of new HCV infection was considerably high. Of the participants in the VIDUS and SEOSI cohorts, 67.9% lacked stable housing at baseline, and HCV incidence was associated with residing in unstable housing in the six months prior to testing HCV positive.

A key finding of the present study is the independent association between residing in unstable housing environments and acquiring hepatitis C. Previous studies have clearly demonstrated that unstable housing among IDU is associated with hazardous and unhygienic injecting practices, such as using drugs in public places, pooling money to buy drugs, and sharing drug-injecting equipment (e.g., needles, spoons) [10], which leads to an increased risk of blood-borne infections [21]. It is likely that the association between unstable housing and HCV incidence is explained by the fact that individuals in unstable housing spend greater time in environments where risk behavior is elevated as a result of the above mechanisms. Past research has also shown that unstable housing is not only associated with poor health outcomes, but also with increased emergency department and hospital service use [22], and as a result the medical costs of homeless individuals in British Columbia are 33% higher per year than those of housed persons [23]. The disorganized lifestyle associated with unstable housing may also act as a barrier for IDU who wish to access primary care and addiction treatment programs. Our study supports this hypothesis, as those with unstable housing were less likely to be involved in a methadone treatment program. As a result, strategies to protect housing for IDU as well as to increase housing for this population, along with interventions that reduce HCV risk behavior among unstable housed individuals, will likely help reduce HCV incidence.

The link between high incidence of HCV and unstable housing is particularly concerning here in Vancouver, Canada, where the number of homeless persons rose by 106% from 2002 to 2005. The number of homeless persons living on the streets of Vancouver's DTES is predicted to exceed 3000 by the time of the 2010 Olympics [23]. Urban renewal in preparation for the 2010 Olympics has led to increased property speculation and increasing property values in the DTES, thereby reducing available low-income housing. The high incidence of HCV infection among local IDU, as well as the risk of HCV infection associated with unstable housing, are indications of the pressing need for both affordable housing and addiction treatment programs. Affordable, stable living environments can decrease hazardous IDU practices that add to the risk of acquiring HCV and, paired with increased access to addiction treatment programs, may decrease the alarming incidence of blood-borne infections such as HCV.

The present study has several limitations. Both VIDUS and SEOSI were created to evaluate risk factors for HIV and HCV transmission but were not developed with the specific aim of evaluating the role of housing. As well, the study design did not allow for determination of the temporal relationship between HCV infection and unstable housing, and although an independent relationship between unstable housing and HCV infection was found, a causal relationship cannot be stated. Specifically, it is possible that risky drug injection behaviour resulted in subjects losing stable housing and that this was associated with, but did not cause new HCV infection. In addition, as a result of limited events and statistical power, we used a combined definition of unstable housing, and future studies should seek to examine the impact of specific unstable housing environments on HCV risk. Qualitative research may also be helpful in better describing how unstable housing environments contribute to HCV risk. There was also a loss of baseline HCV-negative patients to follow-up, which may have influenced results. As well, previous studies of IDU have suggested that socially desirable responding may lead to under-estimation of certain HIV risk behaviors [24], and this likely also pertains to HCV risk factor reporting. However, other studies have suggested self-reports to be valid [25]. While this may have influenced our risk factor analyses, it does not explain the observed differences in both HCV prevalence and incidence. Similarly, studies of marginalized populations may be subject to concerns related to generalizability, as it is not possible to derive a random sample of the overall population. This concern is relevant to all prospective cohort studies of IDU, and it is noteworthy that the demographics of both VIDUS and SEOSI are consistent with what is known about the demographics of the populations of IDU in the local community [14]. A final limitation is that differing recruitment methods were used to derive the VIDUS and SEOSI cohorts. We sought to address this limitation by adjusting for cohort of recruitment in our multivariate model.

## Conclusion

In summary, the present study demonstrates that HCV prevalence and incidence are high among IDU in our setting, and that unstable housing is a risk factor for HCV infection. The current situation here in Vancouver requires an urgent response, including protection of existing low-income housing and increased access to affordable housing. These responses, along with increased access to addiction treatment, may reduce HCV incidence in this setting.

## Competing interests

CK, TK, KL, RZ, MWT and EW have no conflicts of interest to declare. MWT has received research grants from and served as an ad hoc advisor to Tibotec (J&J), BMS, and Gilead Sciences. JSGM has received grants from, served as an ad hoc advisor to, or spoken at events sponsored by Abbott, Argos Therapeutics, Bioject Inc., Boehringer Ingelheim, BMS, Gilead Sciences, GlaxoSmithKline, Hoffmann-La Roche, Janssen-Ortho, Merck Frosst, Panacos, Pfizer, Schering, Serono Inc., TheraTechnologies, Tibotec (J&J), and Trimeris.

## Authors' contributions

CK, TK and EW designed the study. KL and RZ conducted the statistical analyses. CK wrote the first draft and compiled the co-authors' suggestions. All authors have approved the final manuscript. EW had full access to all the data in the study and takes responsibility for the integrity of the data and the accuracy of the data analysis. All authors read and approved the final manuscript.

## Pre-publication history

The pre-publication history for this paper can be accessed here:


